# High-Purity Biomass-Derived Synthetic Graphite: Catalyst-Free Industrial Synthesis and Applications

**DOI:** 10.1021/acsomega.5c09286

**Published:** 2026-01-30

**Authors:** Michal Gulas, Flavie Delort, Raffaele Gilardi, Giovanni Juri, Luca Ostinelli, Frank Rauscher, Xu Wang, Simone Zürcher

**Affiliations:** † IMERYS Graphite & Carbon, Il Centro via Cantonale 65, Bironico 6804, Switzerland; ‡ IMERYS, 43 Quai de Grenelle, Paris 75015, France; § IMERYS Japan, E409 KSP 3-2-1 Sakado, Takatsu-Ku, Kawasaki-City, Kanagawa 213-0012, Japan

## Abstract

To support efforts on the graphite industry’s defossilization, the pursuit of more sustainable options for synthetic graphite might be in contrast with the high requirements for purity, quantity, and quality of graphite materials. The catalyst-free graphitization of pyrolyzed biomass residues into a synthetic graphite is presented. The resulting biomass-derived graphite is characterized by high purity (>99.9%), high crystallinity with a graphitization degree of >91%, and exhibits electrical and thermal conductivity similar to fossil-based synthetic graphites. Material characterization and application data confirm various possible applications, namely in carbon brushes for electric motors and polymer bipolar plates for fuel cells. Since biomass, as called nongraphitizable carbon, is used as a raw material, the graphitization mechanism in the absence of a metal catalyst is proposed and discussed. The reported process and preparation of biomass-derived graphite are confirmed at the industrial scale and thus provide a more sustainable alternative to fossil-based synthetic graphites.

## Graphical abstract

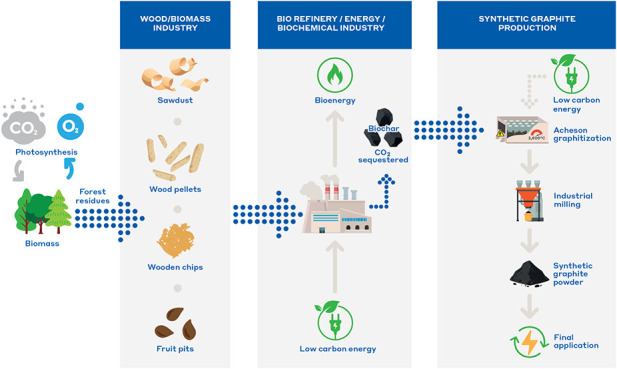

## 1 Introduction

In recent decades, novel carbon materials such as carbon nanotubes or graphene have witnessed a significant acceleration due to their increasing performance and lower production costs and broadened the scope of their applications. However, graphite together with carbon black remains the most produced and used industrial carbon material. This is due, on one hand, to established large-scale processes and the resulting significantly lower cost of production compared to nanocarbons and, on the other hand, to the unique structure and thus better performance in some applications. In particular, graphite, because of its excellent electrical, thermal, and lubricating properties, is commonly used as a functional additive for various applications such as lithium-ion batteries, fuel cells, conductive polymers, brake pads, carbon brushes, and many others.^1–5^

Another advantage of graphite and graphite-like materials is that, over the decades, their physical and chemical properties as well as their main manufacturing methods have been well investigated and understood. It is important to note that the term graphite covers a wide range of degrees of crystallinity and morphologies. Generally speaking, all graphite materials have imperfections in the form of vacancies, dislocations, or stacking faults.^6^ It is commonly accepted that nongraphitic carbons have an interlayer spacing equal to or larger than 0.344 nm, which is the typical value when the carbon structure is nongraphitic, whereas true single crystals of graphite have an interlayer spacing of 0.3354 nm. Intermediate values are observed for partially graphitic carbons with an increasing degree of graphitization.^7^

The annual global production of different graphite materials is around 3 million tonnes with two-thirds being synthetic and one-third natural graphite.^8^ The main synthetic graphite production technology is still the Acheson process, in which the electrical resistance of the carbon precursors is used to increase the temperature to trigger graphitization. The basic principle is described in the patent, “Process of making graphite”.^9^

The most common industrial carbon precursor for the Acheson process is petroleum coke (petcoke), a refinery byproduct produced from the heavy fractions collected in the crude oil distillation and refinery process. Petcoke yield and quality are directly linked to the properties of the crude oil treated in the refinery. It is estimated that the coke yield ranges between 2% and 30% of the amount of crude oil processed. As an example, in U.S. refineries, the average yield is reported to be around 5%.^10^

The production of petcoke has significantly increased in the last two decades, driven by the refining process of heavy crudes and oil sands that tend to have a higher coke yield.^11^ In parallel, the consumption of anode-grade calcined coke, a material from delayed cokers with higher purity and a specific structure used for the electrolytic production of aluminum, has increased due to higher market demand. This demand increase has created tension in the supply chain of high-quality anode-grade petcoke. The driving forces behind the need for different coke qualities are developments and market dynamics for synthetic graphite products, such as the aforementioned graphite anodes for aluminum production, and the global energy transition, which necessitates the adoption of sustainable mobility solutions based on lithium-ion batteries. For some special types of cokes, such as needle cokes, a shortage followed by a dramatic price increase occurred in the past due to both the boom of industrial-scale synthetic graphite production for lithium-ion batteries and the lack of suitable raw materials for its production.

Significant efforts were invested over the years to further improve and optimize the industrial graphitization process, including the efficiency and supply of energy sources as well as raw material selection. However, looking at the various climate legislations and regulations set by different governments around the globe, a step change is needed to achieve those targets.

One of the more probable scenarios to achieve net-zero according to Barre et al.^12^ foresees the phasing out of fossil oil, while simultaneous increasing the use of synthetic graphite; this situation will most likely lead to more stringent supply constraints. Barre highlights alternative options, e.g., biomass-derived graphite from different sources, but points out challenges and limitations in scalability and cost. Similarly, Zhang et al. developed a thorough material flow analysis of graphite from cradle to grave for the U.S. and concluded that graphite recycling^13^ and new raw materials are needed to satisfy graphite demand going forward.

In the past decade, various studies were performed to obtain graphite from biomass.^15–18^ Recently, this research has moved into the spotlight due to the classification of graphite as a critical mineral in the U.S. and EU and increasing export restrictions applied by China. One of the main technical challenges is that biomass-based materials or chars in general are considered nongraphitizable carbons,^14^ which form hard carbons or glassy carbons when exposed to high temperatures.^15,16^ These carbons have distinctly different properties compared to graphite, such as crystallinity and isotropicity.

Banek et al.^17,18^ demonstrated the synthesis of graphite and nongraphitized carbons for mobile energy applications from hardwood sawdust and cellulose spheroids using irradiation with diodes and a CO_2_ laser. Shi et al. and Molaiyan et al.^19,20^ confirmed possible applications of biobased graphites as active anode material for lithium-ion batteries, but also other applications like inks and pencil leads.^21^ All these synthesis methods have in common the use of a catalyst^22,23^ and therefore require a subsequent purification step for the obtained product. This requires rather a high use of chemicals (1–1.5 g/g of biomass), although some are recyclable, and the use of water in the range of 10–20 L/kg of biomass is needed.^24^ The purification process is usually done by acid washing using HNO_3_ and HCl, and it is also energy- and resource-intensive and therefore not preferred. Since iron-based catalysts are mainly used, purification is critical, especially for applications such as lithium-ion batteries or fuel cells where extremely high-purity carbon-based materials are required.^25,26,27^

Wang et al.^28^ prepared a graphitic material without the use of a catalyst, by a high-temperature and high-pressure method; however the carbon content of the as-prepared sample was only as high as 90% and with crystallites size only at 50 nm. Yap and coworkers^29^ discuss recent advances in the production of graphite from lignocellulose-based biomaterials. It is interesting to note that Yap discusses the advantages of the Joule heating process to prepare low-cost but high-conductivity graphitic carbons, citing examples for producing graphitic lignin carbon from reduced graphene oxide-lignin^30^ and carbon fibers from bamboo.^31^

Ike and Vander Wal,^32^ in a recent study, demonstrated the importance of volatiles and devolatilization during the graphitization of hard carbons. Employing pressurized carbonization, volatiles could not escape and attach to the edge sites of graphitic structures, where they increase the crystal growth. However, even with this method, they could not obtain fully graphitic materials, as shown in their study.

Cherakkara et al. and Nzihou et al.,^24,33^ in their recently published reviews, highlighted that direct graphitization without a catalyst does not provide the required quality and quantity of graphitic domains and necessitates specific pretreatment. However, adding further process steps, such as hydrothermal carbonization leads to a high energy consumption and thus potential issues for future industrial application.^32^ Cherakkara, besides the already mentioned catalytic graphitization, discusses advanced techniques to produce synthetic graphite from biomass such as a microwave-assisted process, flash joule heating, and ultrasonic methods. However, all these methods come with disadvantages, such as the higher number of process steps, process control issues, process complexity, and obtained material quality, which makes the implementation of these processes challenging for large-scale synthesis. In a very recent review, Bhattacharyya et al.^34^ discuss the need for innovation in graphite production as a critical mineral. The criticality arises mainly from the need for high purity of structurally ordered carbon material. The review also notes that the Acheson process is best-in-class for three out of five parameters, while noting the sustainability shortcomings of the Acheson process. This point is addressed by the results presented here—the production of green graphite on an industrial scale.

In this study, a high-purity, highly crystalline, and isotropic biomass-derived synthetic graphite (BDSG) is produced from different biosources using the Acheson process. The advantages of this process technology are its large industrial scale, high efficiency, and the fact that no catalyst is needed. According to our knowledge, this is the first time that high-crystallinity carbons (i.e., synthetic graphite) are produced at an industrial scale using biomass residues as raw materials without the use of a catalyst and any additional postpurification process. In particular, the avoidance of a catalyst and subsequent acid washing favors this process, as it eliminates steps where aggressive chemicals are used. Additionally, this process is also discussed from a sustainability perspective, and the methodology for Life Cycle Assessment (LCA) of biomass-derived graphite is discussed.

Using the Acheson technology,^4^ a well-established process that delivers Joule heating, provides the possibility to employ a proven technology to offer industrially produced materials as an alternative to coke-based synthetic graphite for many applications at relevant industrial quantities.

## 2 Materials

Different types of biobased raw materials were used as starting materials in this study. Samples of biocarbons and chars, made from different biomass waste streams, were graphitized and underwent postprocessing as described in Figure 1. Pyrolyzed biomass residues or byproducts used in this study can all be described as high-carbon-content byproducts of biomass pyrolysis. The char properties can vary based on the source of biomass as well as on the pyrolysis process used (see Table 1). Ultimately, the source and process define the biocarbon composition, the amount of volatiles, its density, and the carbon solid content.

**Figure 1:**
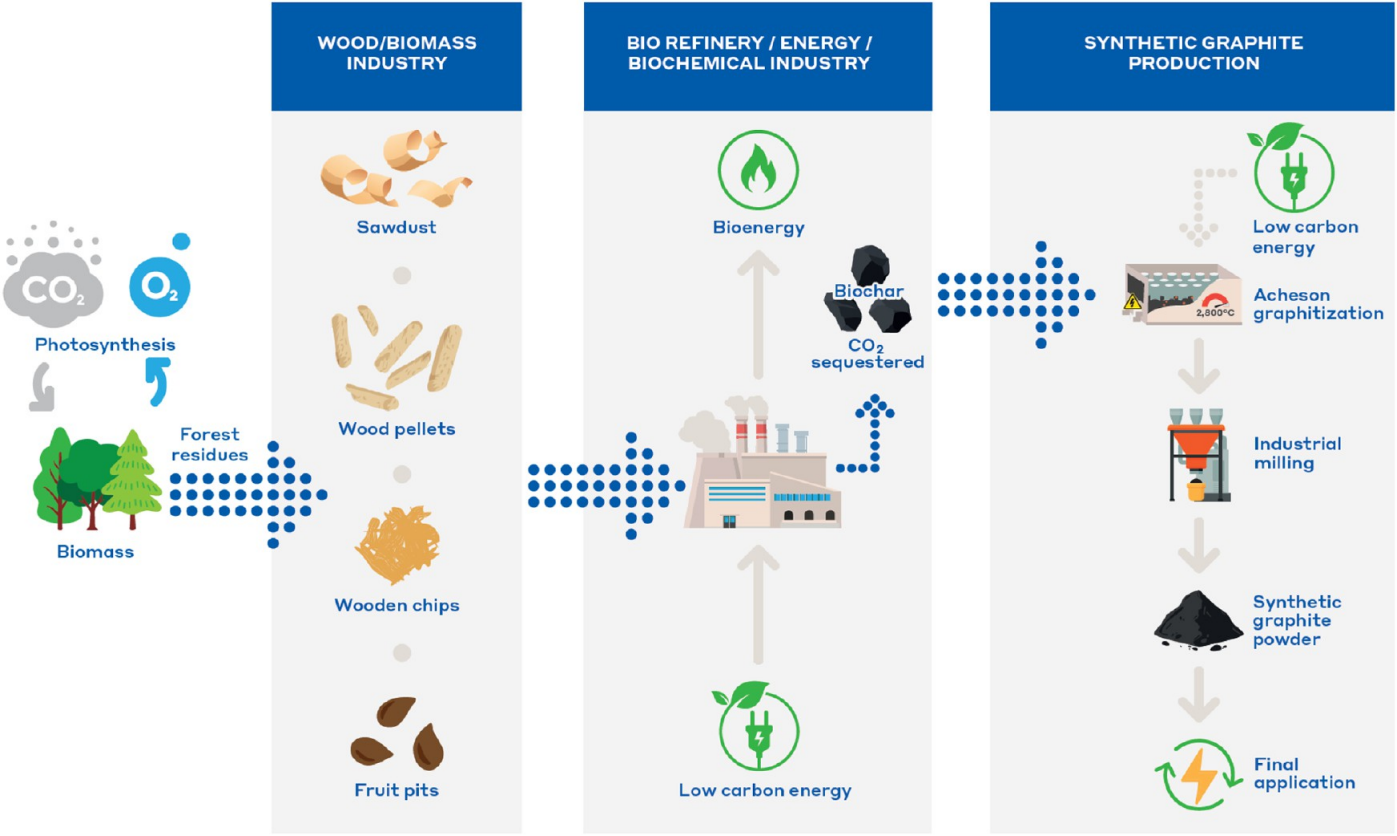
General process steps of biomass-derived synthetic graphite production: from biomass and various forest residues through pyrolysis, graphitization, and milling to final application.

**Table 1:** Properties of Biomass-Based Raw Materials Used for BDS Graphite Synthesis in This Study^a^

					Vibrating sieving [%]	
Biomass based raw material	Biomaterial source	Ash [wt %]	Volatiles [wt %]	Bulk density [g/cm^3^]	>2 mm	>250 μm
**A**	**Charcoal from wood residues**	1.7	10.3	0.15	0.0	56.8
**B**	**Biochar made from wood chips sieving residue**	8.0	18.1	0.13	39.7	89.0
**C**	**Biochar based on forestry and wood industry residues (waste wood chip and pellet, fruit pits)**	2	11	-	57.6	92.1
**D**	**Biocarbon from pine sawdust**	12.3	7.3	0.09	4.3	75.5
**E**	**Biocarbon based on forestry and wood industry residues (sawdust, wooden pellets, fruit pits)**	1.8	7	-	0	23.2

a For clarity and simplicity reasons, materials produced using C and E are not discussed in Section 4. However, results also for these two sources confirm the same trend and are available upon request. The scope of listing it here is to show that various biomass sources can be used.

## 3 Synthesis

The major process steps needed to convert biomass into a biomass-derived graphite material are shown in Figure 1. This study focuses on the conversion step of pyrolyzed biomass into graphite, considering various biobased materials as feedstock. In our case, the graphitization step was carried out using graphite crucibles placed on the center line of an Acheson furnace to undergo a standard graphitization step. The graphitization cycle is characterized by a dwell time of about 24 h at a temperature range of 2800–3000 °C and a subsequent cooling phase.

Biomass-based materials with properties listed in Table 1 were loaded into the graphite crucibles with a volume of 8.5 L. The filling ratio of these boxes was usually between 80% and 90%, resulting in a loading of around 1000–2000 g depending on the density of the raw material. No other material was loaded into the box, resulting in 100% biomass-based raw material.

Since the total carbon content (total mass minus ash) of the raw materials is typically higher than 90% (see Table 1), the final yield was in the range of about 80% with the main loss due to partial volatile release and moisture (typically ∼10%). For example, in the case of BDSG B (listed in Table 2), the loading amount was 1550 g per box and the material output was 1190 g.

**Table 2:** Properties of Biomass-Derived Graphite (Based on Biomass Sources from Table 1) and Reference Synthetic Graphites (Coke-Based) with Different Particle Size Distributions Required for Different Applications

					Particle Size Distribution					
	SSA [m^2^/g]	Xylene Dens. [g/cm^3^]	Scott Dens. [g/cm^3^]	Springback [%]	d10 [μm]	d50 [μm]	d90 [μm]	Lc (002) [nm]	c/2 (002) [nm]	g [%]
**Graphite GC (coal based)**	10.5	2.108	0.15	31	5.2	18.8	43.6	105	0.3359	94
**Graphite GGC (coke based)**	9	2.259	0.16	31	3.6	12.8	30.5	68	0.3363	90
**TIMREX KS 5-75TT**	4	2.248	0.45	21	20.3	44.1	88.5	170	0.3358	95
**TIMREX KS75**	7.2	2.253	0.23	14	5	23	67	174	0.3358	95
**BDSG A**	6.7	2.065	0.14	42	2.9	14.5	76.8	100	0.3359	94
**BDSG B**	7.6	2.117	0.15	37	2.9	12.9	36.6	101	0.3359	94
**BDSG D**	9.2	2.111	0.16	38	4.2	14.4	37.1	111	0.3358	95
**BDSG D25.1**	12.8	2.205	0.13	29	3.3	9.2	21.0	91	0.3359	94
**BDSG D25.2**	12.9	2.184	0.13	28	3.0	9.2	23.0	95	0.3359	94
**BDSG D75**	-	2.130	0.23	36	2.2	13.0	73.5	96	0.3358	94

The oxygen-free treatment atmosphere, as will be discussed in Section 5.2, is mainly composed of volatiles released from the biocarbon samples.

After recovering the as-synthesized graphite from the crucible, the coarse raw graphite is first processed to break up larger lumps into centimeter-sized particles and subsequently milled to different particle size distributions (PSD) in the range of 10 to 75 μm (d90). The milling step was done using different milling technologies like air jet milling or hammer milling, which results in graphite particles with different specific surface areas, shapes, and surface functionalization groups.^35,36^ The whole process is carried out with special precautions to avoid contamination of the high-purity synthetic graphite samples (5–10 kg) discharged from the crucibles.

In the Acheson furnace, thermal energy is produced by the passage of an electric current through a central core or through the loaded material itself. The charge does not melt and remains nearly in the same position until the end of the graphitization step.^37^ In his handbook, Stansfield mentioned that the Acheson process is typically used for two ways of graphite production: (i) the conversion of anthracite into the bulk graphite and (ii) the graphitization of electrodes. The former process, with its modifications regarding yield, efficiency, automation, and scale, is employed in this study. In the past, as Stansfield states, anthracite was selected as the most suitable raw material for graphite production; however, due to its poor electrical conductivity at low temperatures, there is a need to use a core-carbon rod that carries the current. Generally, the Acheson process allows the preparation of high-purity graphitized materials because impurities from the raw materials migrate to the outer layers during the graphitization step and act as carbide-forming materials. The purification process is driven by the melting, sublimation, or decomposition of the different materials and their subsequent transport to the cooler zone.

Biomass-based materials (e.g., biochar or char) are generally considered to be nongraphitizable carbons. Exposing char to temperatures above 1500 °C typically leads to the formation of hard carbon and further conversion to glassy carbon occurs above 2500 °C. This was observed and reported in many studies.^38,39,40,41,42^ Glass-like carbons are defined as materials with high porosity, low crystallinity, and very high isotropy in their structural and physical properties.^14^ As mentioned by Li,^43^ carbon materials with a higher graphitization degree are difficult to prepare from hard carbons.

Contrary to this, we observed that by using Acheson technology, graphitic materials with high crystallinity and a graphitization degree (small interlayer c/2 distance) and rather high isotropy can be produced, as further discussed in the sections below.

Given that the yield of biocarbon conversion to synthetic graphite is similar to that of fossil-based raw materials, the primary cost difference between BDSG and fossil-based synthetic graphite lies in the cost of raw materials. It is believed that the cost differences between fossil-based raw materials vs biomass-based raw materials are for the moment mainly driven by the differences in the economy of scale. Biochar production units still have rather small capacities per unit (<5000 t/year) but with a predicted market growth rate for the biochar industry at 50% Compound Annual Growth Rate (CAGR) by 2030.^44^ This growth, mainly motivated by the fact that biocarbons are considered the most relevant CO_2_ removal industrial option for permanent carbon removal in Europe and globally,^45,46^ will lead to capacity increase and price decrease. On the other hand, the price of fossil-based raw materials like cokes will in the long term increase due to the stringent emission targets. Considering the possibility of CO_2_ credits being applied to biomass-based materials, it is believed that in the long term (within a 10-year horizon), the costs of biomass-based materials will align with those of fossil-based materials. This leads to the conclusion that the future production costs of BDSG will be in the same range as conventional fossil-based synthetic graphite.

## 4 Results

As anticipated in the introduction, in the following section, we discuss the results obtained for synthetic graphite materials based on biomass feedstock. Analytical techniques such as X-ray diffraction and Raman spectroscopy, discussed in more detail below, clearly confirm the graphitic structure of the produced materials. With the support of visualization techniques such as scanning electron microscopy and polarized light microscopy, high aspect-ratio structures with larger graphitic domains can be observed.

The material properties of different graphitized biomass-based materials together with typical synthetic graphites based on coke as reference samples are given in Table 2. Furthermore, the impurity levels of selected biomass-derived graphite samples are provided in Table 3. Nitrogen absorption specific surface area (SSA) shows values very similar to the reference synthetic graphite for otherwise comparable properties. The results using nitrogen gas also confirmed porosities similar to the reference synthetic graphite and unlike hard carbons, as explained by an extensive study performed by Ghimbeu et al.,^38^ where N_2_, Kr, and CO_2_ were used to assess the porosity of hard carbons. The minor differences between SSA shown in Table 2 can be explained (even though this is not the purpose of this study) by slight differences in the particle size distribution of the analyzed samples as well as by different milling techniques used, which result in different amounts of surface functionalization groups.

**Table 3:** Selected Biomass-Derived Synthetic Graphites (BDSG) Showing High Purity Levels of Selected Elements

	Ash [%]	Fe [ppm]	Ni [ppm]	Cr [ppm]	Al [ppm]	S [ppm]	Si [ppm]	Na [ppm]	V [ppm]
**BDSG A**	-	67.6	5.1	2.3	12	1.8	62.1	9.9	8.7
**BDSG B**	-	38	0.7	0.9	10.2	0	18.4	4	4.8
**BDSG D**	0.09	32	0.9	0.9	9.7	0	14.7	3.4	6
**BDSG D25.1**	-	54.2	1.5	5.5	15.5	0	66.8	0	23.6
**BDSG D25.2**	0.008	15	0.7	1.2	3.8	8.7	49.7	2.6	14.4

X-ray data were collected using a Malvern Panalytical Empyrean diffractometer coupled with a Malvern Panalytical Pixcel-3D Medipix3 detector and using a Cu X-ray tube with monochromators. The diffraction patterns were analyzed according to the standard procedure of X-ray measurements on carbon materials, as described by Iwashita et al.^47^ The degree of graphitization *g* is calculated with the formula suggested by Seehra and Pavlovic^48^ and is directly related to the interlayer spacing (*d*_002_) by

$$g=\frac{\left(0.3440-d_{002}\right)}{\left(0.3440-0.3354\right)}$$

As can be seen in Table 2, the graphitization degree of the graphite samples from biomass is very high (over 90%) and comparable to that of reference synthetic graphite from coke. The high crystallinity is also evident by looking at the diffraction diagram of the different graphites (Figure 2).

**Table 4:** Ratio of Peak Heights and Their Areas after Deconvolution and Corresponding *L_a_* Calculated Based on References from Cancado et al. and Tunistra et al., Respectively

	*I*_G_/*I*_D_	*L*_a1_ [nm]	*A*_G_/*A*_D_	*L*_a2_ [nm]
**TIMREX KS**	3.9	23	1.2	47
**BDSG D**	5.6	33	1.8	70

**Figure 2:**
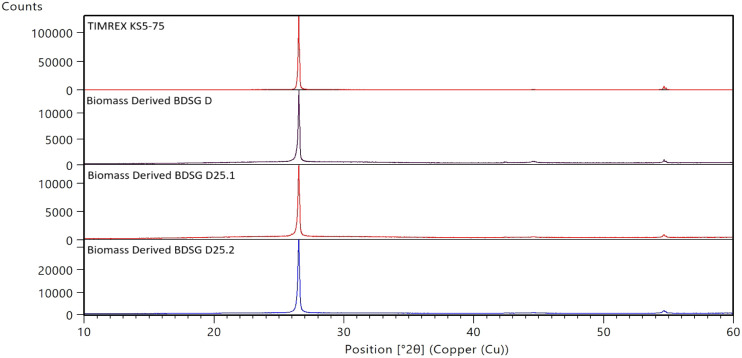
X-ray diffraction diagram of BDSG biomass-derived graphites and reference synthetic graphites (coke-based) showing typical [002] peaks and confirming high sample crystallinity (>90 nm).

However, the size of the crystalline domains of the graphitized biocarbons, *L_c_* (002) calculated from the fwhm of the [002] peak according to Iwashita,^47^ differs significantly from that of the coke-based synthetic graphites at the similar particle size: 170 nm for KS5-75 vs 96 nm for BDSG D75 (see Table 2). This difference in the crystallinity can also be visually recognized in the diffraction profiles of the [004] peak of the different graphites in Figure 3 below, where the peak of TIMREX KS5-75 is narrower and better defined compared to that of the biomass-derived graphites.

**Figure 3:**
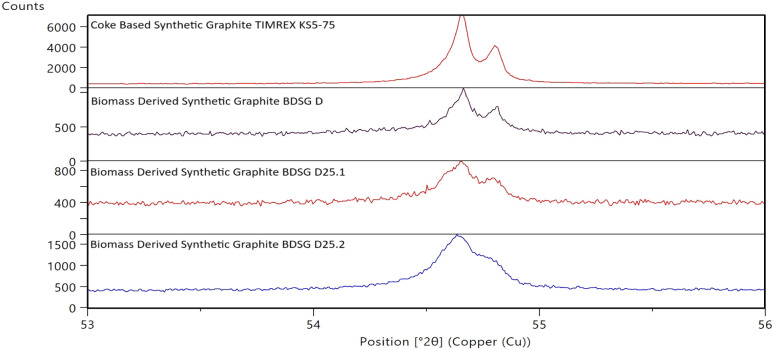
Diffraction profiles of the [004] peak of biomass-derived synthetic graphites BDSG and reference synthetic graphites (coke-based).

Raman analysis was performed using a LabRAM-ARAMIS Micro-Raman spectrometer from HORIBA Scientific with a 633 nm laser at room temperature. The *I*_G_/*I*_D_ ratio (“*R* value”) is based on the ratio of intensities of the so-called band D and band G. These peaks are measured at 1350 cm^–1^ and 1580 cm^–1^, respectively, and are characteristic of carbon materials. Raman spectroscopy is a complementary method to XRD; it identifies surface crystallinity because the penetration and detection depths are in the range of hundreds of nanometers, depending on the laser wavelength. This allows for providing additional information about biomass-derived graphite samples and their comparison with reference synthetic graphite. Table 4 shows the ratio of intensities (height) of band G representing structural order and band D indicating the degree of disorder (*I*_G_/*I*_D_), as well as their areas (*A*_G_/*A*_D_). Looking at the ratios, it is confirmed that surface crystallinity is higher or in the same range as synthetic graphite. For this measurement, synthetic graphite samples with *L_c_* = 160 nm and with a similar particle size distribution of D_90_ 40–50 μm were chosen to exclude potential differences due to the milling process.

Based on the references from Tuinstra et al. and Cancado et al.,^49,50,51,52^ the average crystallinity in the *a* direction (*L_a_*) can be calculated based on the intensity ratios and area ratios, respectively. These values correspond to 23 and 47 nm, respectively, for synthetic graphite and 33 and 70 nm respectively for BDSGs.

The quality of the deconvolution of the measured spectra, after background subtraction, as well as their fit using Lorentzian profiles, is shown in Figure 4. Besides the already mentioned band D and band G, band D′ was also added. The spectra were normalized to the height of the G band for easier visualization, which also helps to observe differences between coke-based synthetic graphite and biomass-derived graphite.

**Figure 4:**
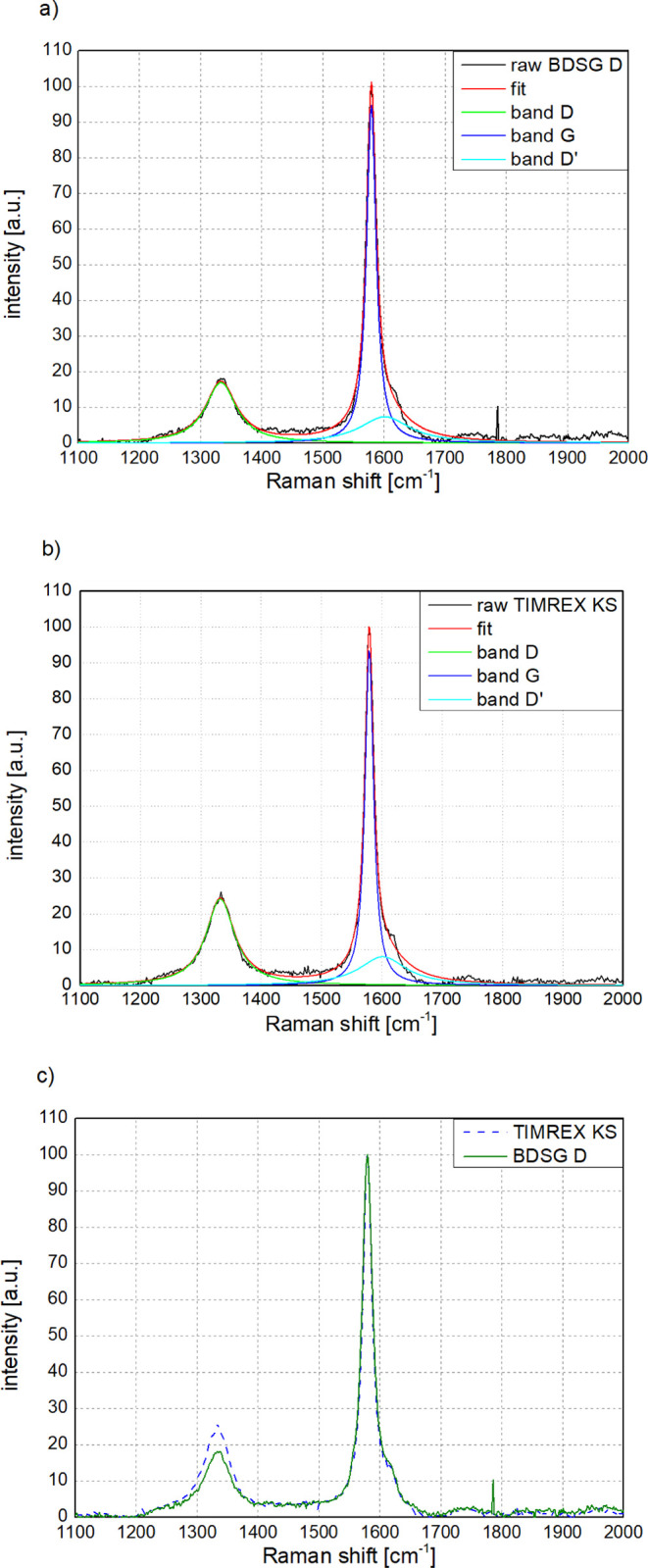
Raman spectra of graphites showing typical D and G bands. a) Deconvolution of Raman spectra of biomass-derived graphite D into bands D, G, and D′; b) Deconvolution of TIMREX KS graphite used for calculating *L_a_* shown in Table 4. c) Superposition of the two materials normalized to 100 indicating significantly lower intensity of the D band (∼1350 cm^–1^) due to higher crystallinity in the *a*-direction (*L_a_*) for BDSG graphites.

Scanning Electron Microscope (SEM) analyses shown in Figures 5–7 revealed rather isotropic structures compared to typical coke-based synthetic graphite (without any preconditioning like agglomeration of fines or spheroidization). Under a closer look with a higher magnification, typical high aspect-ratio structures for flaky graphite can be found (Figure 7), which correspond to the Raman and XRD observations. At the same time, structures more typical for hard carbon—highly porous materials—are also present (Figure 5 left).

**Figure 5:**
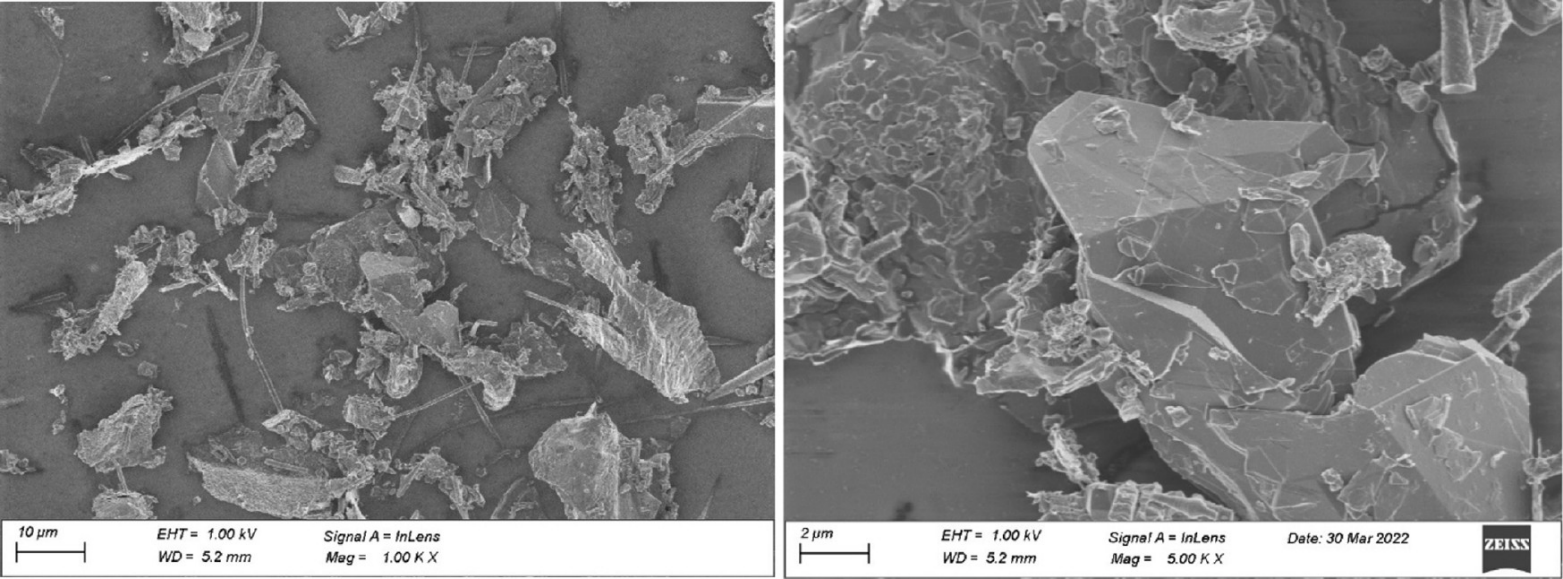
SEM images of biomass-derived graphite BDSG A show typical structures for hard carbons (left) but also isotropic graphite particles (right).

**6:**
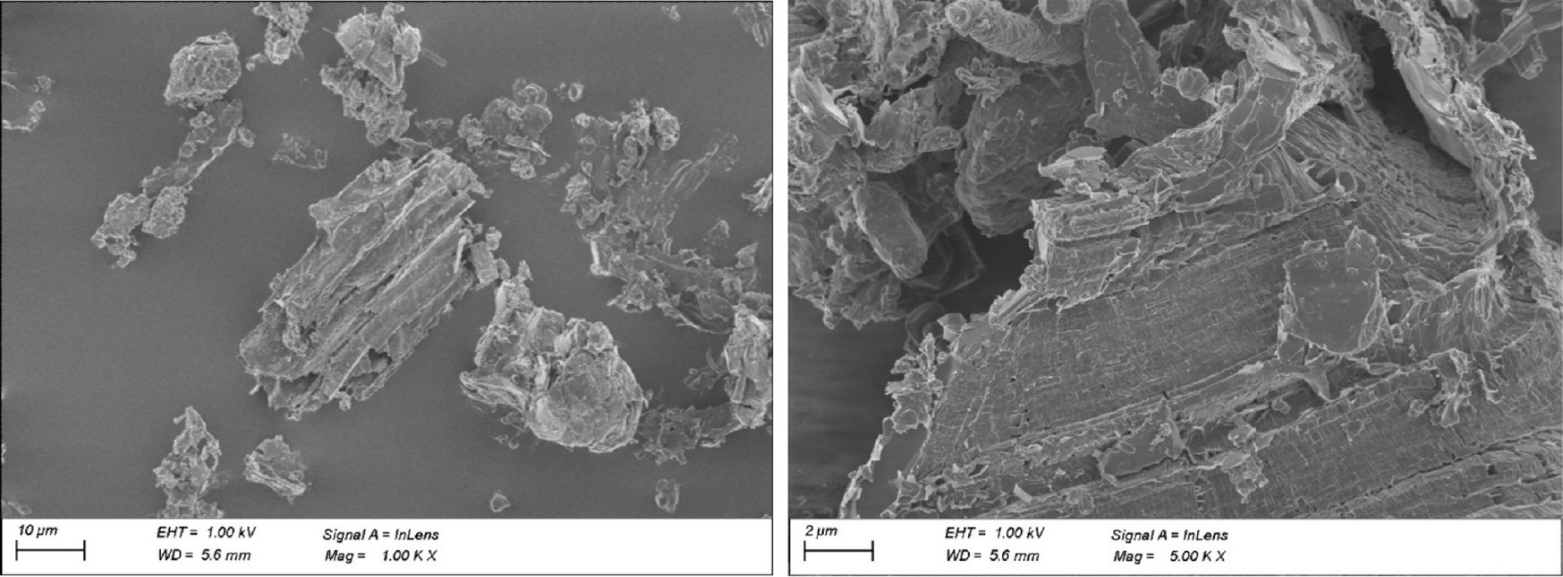
SEM images of BDSG B showing typical flaky structures, however, with more isotropic features compared to coke-based synthetic graphite.

**Figure 7:**
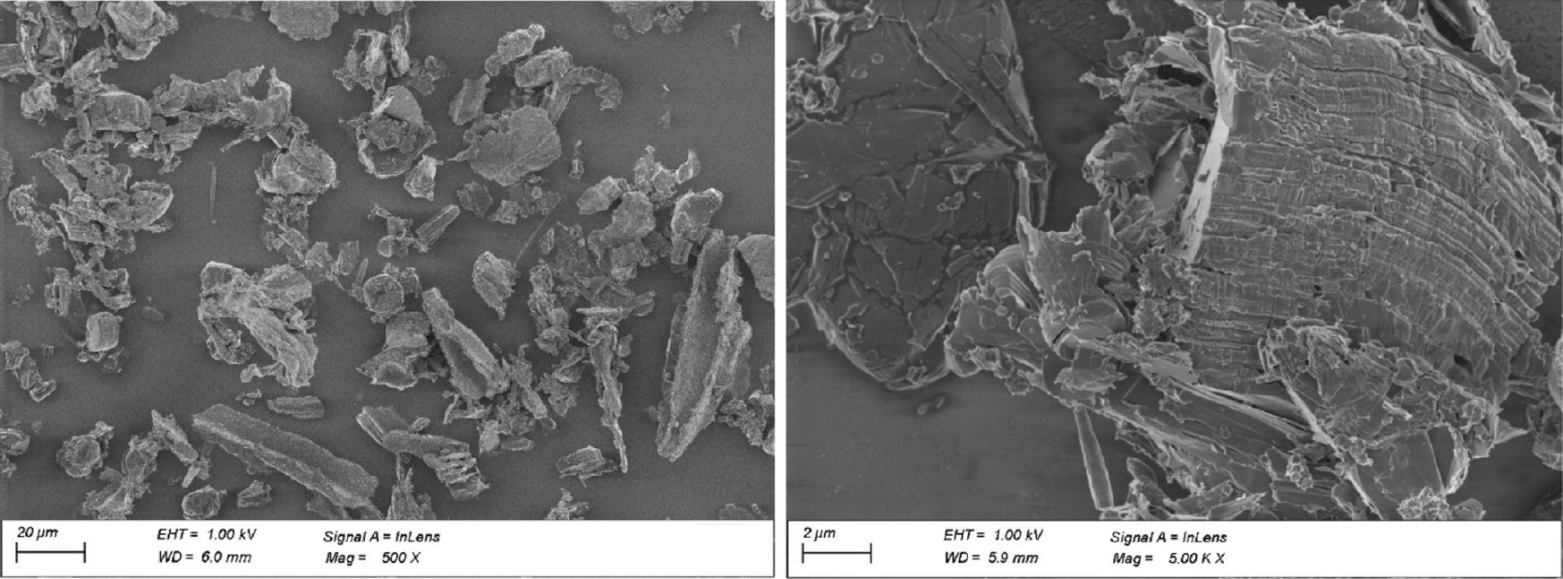
SEM images of BDSG D showing high aspect ratio particles.

Polarized light microscopy is a powerful imaging technique to visualize the optical domain size and texture. This can help in understanding the influence of the raw material and its porosity on the graphitization level from a microscopic point of view. Rørvik et al.^53^ developed an automatic method for image analysis of the optical texture of cokes. As described in the above-mentioned reference, bireflectance of the analyzed grains provides interference colors depending on the local direction of the graphitic layers. Samples were prepared and analyzed according to the cited reference; however, an automatic mode to determine mosaic and fiber index was not employed due to the lack of crystalline domains in the raw biomaterials.

From optical images (nonpolarized and polarized light) shown in Figure 8, one can see that pyrolyzed biomass or forest residues (Figure 8, left side), as expected, are materials with high internal porosity and typical 2D fused rings.^54^ However, samples analyzed after graphitization (right) show more graphite-like structures, but some grains with higher porosity can still be found. In polarized light pictures one can see that optical domains start to have sharper boundaries compared to the heat-treated biomass-based material and coke as well.^53^ While in biocarbon, the optical domains are hardly distinguishable, the graphitized samples are well defined. It is also worth mentioning that in the polarized light analysis of graphitized material, grains with higher porosity can still be identified.

**Figure 8:**
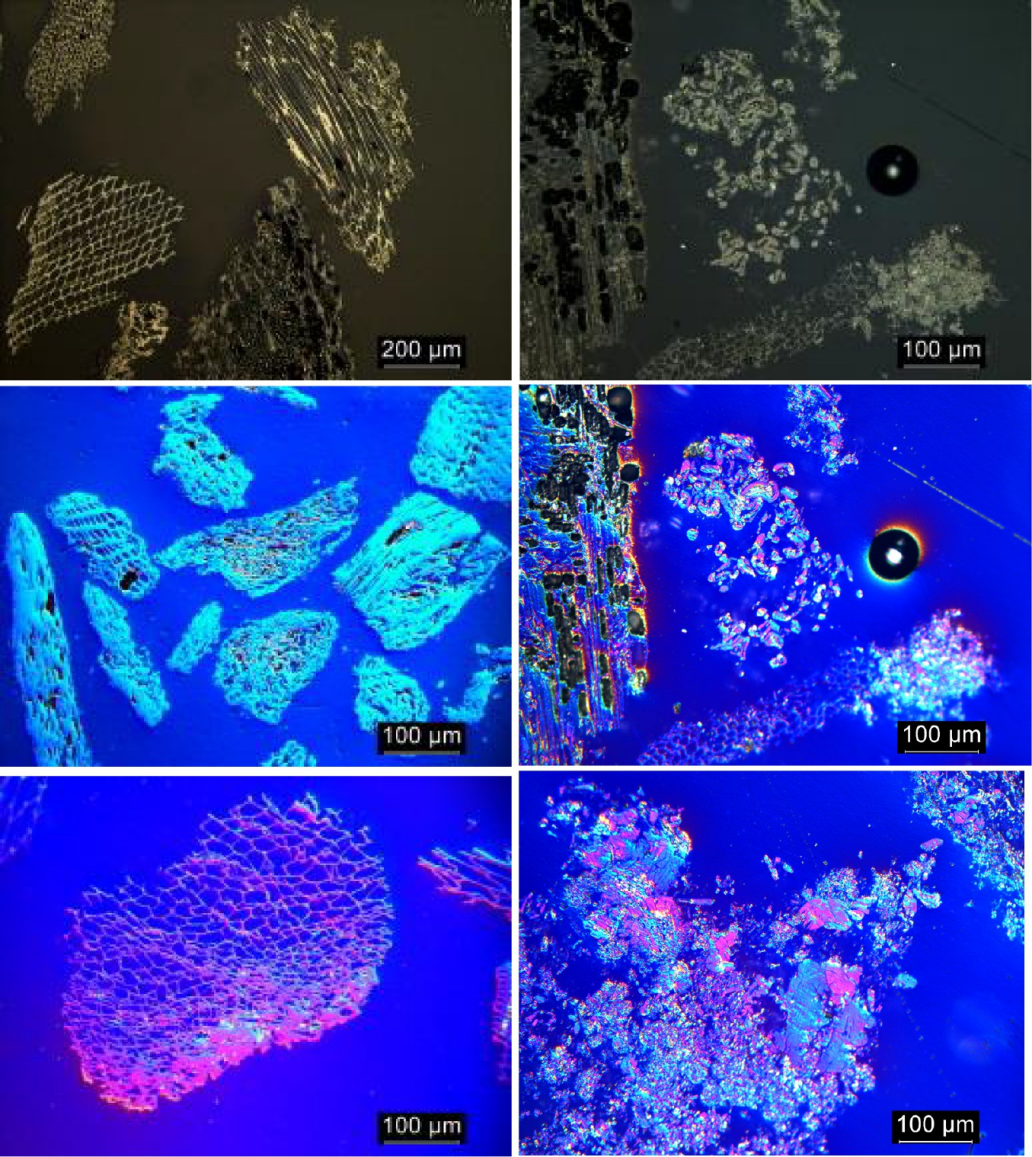
Nonpolarized and polarized light microscopy images showing biomass-based feedstock B before graphitization (left) and biomass-derived graphite BDSG B (right).

As a comparison, the same method was used for petroleum coke and synthetic graphite made from petroleum coke shown in Figure 9 (left and right, respectively). The difference between pyrolyzed wood chips and petcoke is evident. Petcoke shows no porosity and a higher structural order, as already observed by Rørvik et al.^53^ Graphitized coke, on the other hand, as expected, does not contain any high-porosity structures and shows very high anisotropy compared with biomass-derived synthetic graphite.

**Figure 9:**
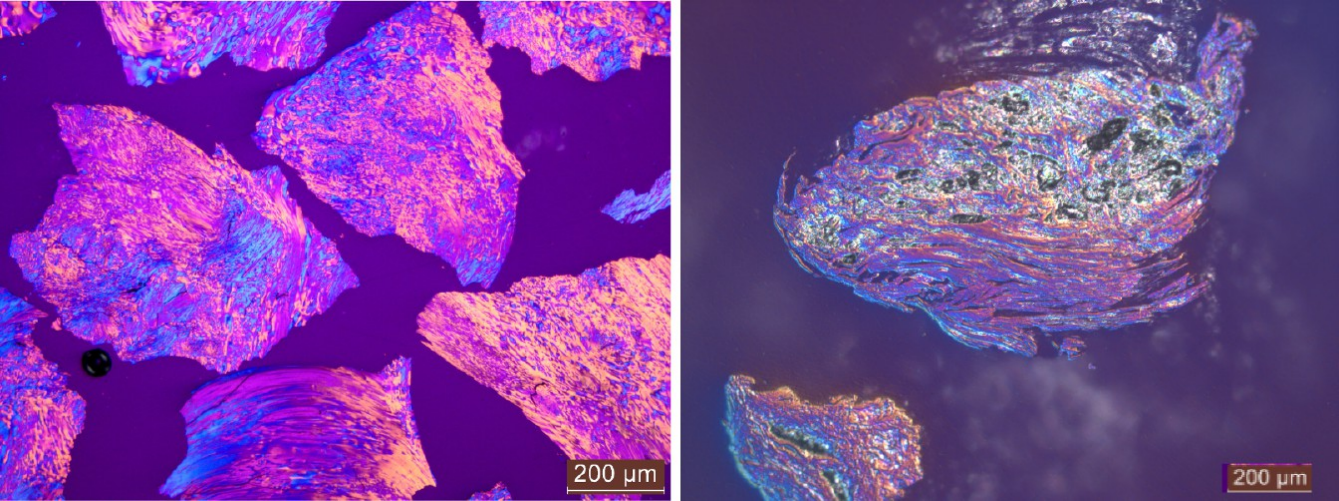
Polarized light microscopy images showing petroleum coke (left) and the corresponding synthetic graphite (coke-based) (right).

A certain amount of closed internal porosity in graphitized biochar samples is also confirmed by lower real (xylene) density values. As seen in Table 2, graphitized biomass-derived graphite D shows a real density of 2.11 g/cm^3^ with an average particle size of 14 μm compared to coke-based synthetic graphite TIMREX KS75 with a real density of 2.25 g/cm^3^ for 23 μm particles. It is worth noting that this closed internal porosity is further opened when particles are milled down to an average size of 9 μm. This can be seen in BDSG D25.1 and BDSG D25.2 with real densities of 2.2 and 2.18 g/cm^3^, respectively. These biomass-derived graphites are from the same batch of biomass source and were produced using the same graphitization parameters, with the only difference being the milling step.

Purity levels of the as-prepared BDSG were analyzed by Spark Discharge Optical Emission Spectroscopy (SD-OES) and are summarized in Table 3. As shown by the data, exceptional purity without any additional purification step is obtained. Iron levels in the range of 15–70 ppm, Ni and Cr from 1 to 5 ppm, and other metals depend not only on the raw material but also on the type of granulometry and milling process selected. Elements like sulfur and sodium, typical for pyrolyzed biomass sources, are at very low levels, especially compared to catalytic graphitization. It is worth noting that the level of vanadium, a typical trace element for synthetic graphite, is also low. These findings correspond with the observation that the ash level of carbonized biomass (listed in Table 1), in the range 1–12%, decreased after graphitization to 0.01–0.1%.

As-prepared biomass-derived synthetic graphite has some distinctive features compared to catalytically produced biobased graphites. Shi et al.^19^ present catalytic graphitization using hybrid catalysts; however, they reached a lower graphitization degree (89%) and similar yield (74%) compared to the present study. Jabarullah^55^ prepared biographite from palm waste using iron and nickel catalysts, which resulted in materials with lower crystallinity of around 60 nm. Dey^56^ catalytically graphitized pyrolysis oil, which resulted in biographite with significantly higher iron content (500 ppm) even after acid washing. And You et al.^57^ prepared high-crystallinity graphites, similar to natural graphite, and confirmed their application in inks.

The BDSG biobased graphites have been tested for different applications, specifically carbon brushes for electric motors and polymer bipolar plates for fuel cells. An initial feasibility study also showed the potential as an anode active material in lithium-ion batteries and other applications, such as brake pads and powder metallurgy.

### 4.1 Application Tests in Carbon Brushes

A carbon brush has the primary function of delivering or collecting current from a rotating part of an electric motor. Therefore, low electrical resistivity is required. Typical carbon brushes for household appliances are composed of 80 wt % of synthetic graphite and 20 wt % of phenolic resin (which acts as a binder). As part of this study, we tested the new biomass-derived graphite BDSG vs conventional synthetic graphite in carbon brushes and compared the application performance.

As shown in Figures 10 and 11, carbon brushes with biomass-derived graphite have lower density compared to coke-based synthetic graphite due to the higher spring-back (see Table 2). However, carbon brushes with BDSG also exhibit a low electrical resistivity, confirming that these biomass-derived graphite samples have a high degree of graphitization and therefore good electrical conductivity. It is worth noting that the ratio between through-plane resistivity and in-plane resistivity (the so-called electrical resistivity anisotropy), shown in Figure 12, is much lower for BDSG compared to standard synthetic graphites. This is a clear indication of the more isotropic properties of biomass-derived graphite.

**Figure 10:**
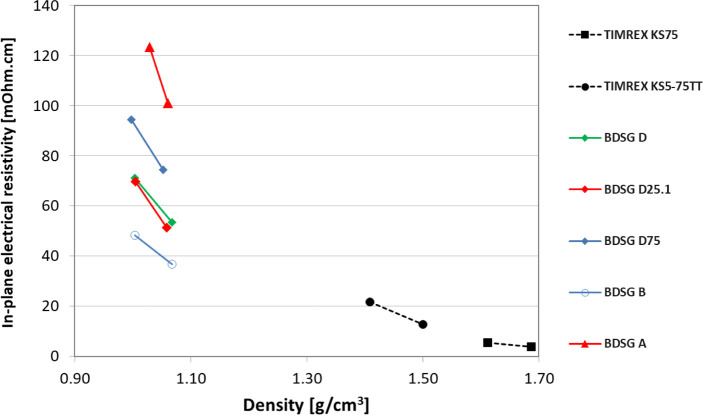
Biomass-derived synthetic graphites (BDSG) compared to coke-based synthetic graphites have a lower density while maintaining low in-plane electrical resistivity.

**Figure 11:**
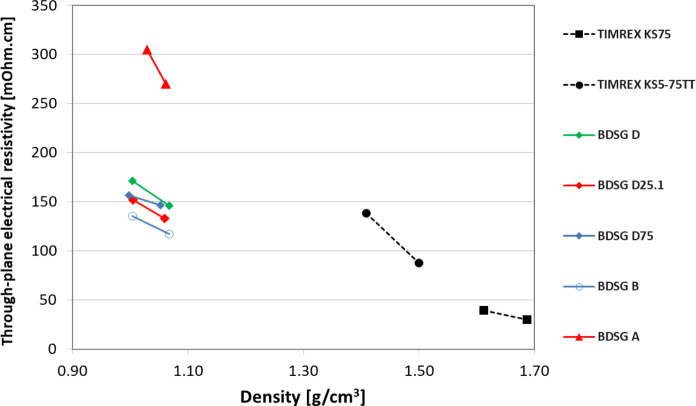
Biomass-derived synthetic graphites (BDSG) compared to coke-based synthetic graphites have lower density while having similar through-plane electrical resistivity and therefore good electrical conductivity.

**Figure 12:**
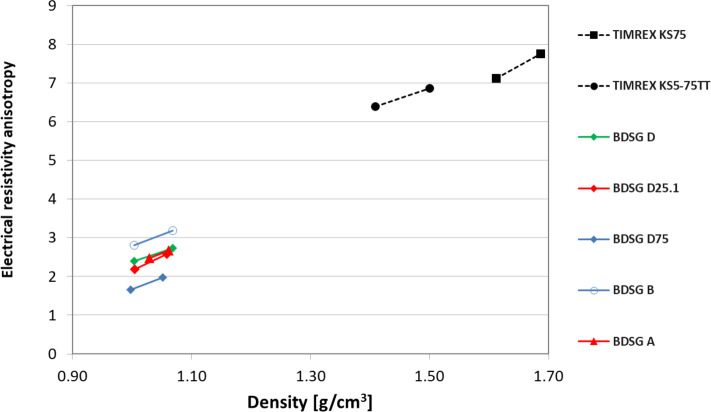
The isotropic nature of biomass-derived synthetic graphites, expressed as the ratio between through-plane resistivity and in-plane resistivity (electrical resistivity anisotropy), is much lower for BDSG compared to standard synthetic graphites, indicating more isotropic properties.

Overall, the use of BDSG in carbon brush applications is feasible if isotropic electrical resistivity is needed and mechanical property requirements are low. Carbon brushes that require isotropic electrical resistivity are typically those used in applications where uniform current distribution in all directions is required, such as high-speed electric motors and large power generation systems. Adaptations of the formulation (e.g., percentage of graphite vs percentage of resin), type of resin, and combination of different graphites in the same formulation are mandatory to find the best performance depending on the application requirements.

### 4.2 Application Tests in Polymer Bipolar Plates for Fuel Cells

Polymer bipolar plates are key components in proton-exchange membrane fuel cell (PEMFC) systems. One of the main functions of bipolar plates is to conduct the electrical current from cell to cell. Therefore, low electrical resistivity is required, in particular in the “through-plane” direction.^58^ We tested the BDSG-based samples and compared their properties against coke-based synthetic graphite.

As shown in Figure 13, bipolar plate samples containing BDSG show electrical resistivity similar to that of the ones made with coke-based synthetic graphite, in some cases even lower. In conclusion, biomass-derived graphites could also be used for this application, although further properties (such as permeability, mechanical properties, and so on) will require further evaluation and investigation.

**Figure 13:**
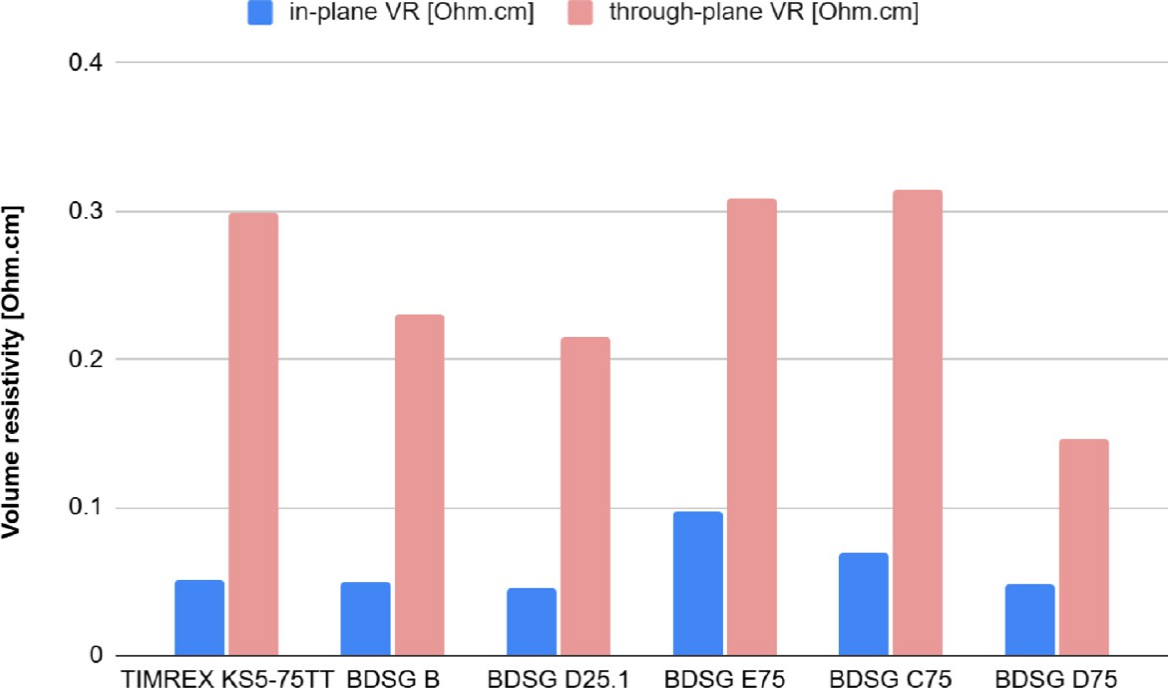
Bipolar plate samples containing biomass-derived graphites show electrical resistivity similar to that of coke-based synthetic graphite. In some cases (e.g., BDSG D25.1 and D75) even higher conductivity.

### 4.3 Application Tests as an Anode Active Material for Lithium-Ion Batteries

Apart from the above-mentioned applications, biomass-derived synthetic graphite BDSG is further explored as an active anode material in an anode electrode for lithium-ion batteries.

The coin cell test results of two BDSG samples compared to those of a coke-based synthetic graphite, are shown in Table 5. Despite the fact that the biomass-derived feedstock has a lower carbon content vs coke-based synthetic graphite’s raw material, the biomass-based graphites show a reasonably high reversible lithium-ion storage capacity of over 330 mAh/g. This corresponds to the high degree of graphitization of biomass-derived graphite as confirmed by XRD. However, due to the high BET surface area of the as-prepared BDSGs, the initial Coulombic efficiencies were lower than the commercial synthetic graphite, showing an initial irreversible capacity of 47–48 mAh/g. Further amorphous carbon coating can help to reduce the BET surface area and further improve the Coulombic efficiency.

**Table 5:** Coin Cell Test Results of Two Biomass-Derived Graphites BDSG vs Reference of Coke-Based Synthetic Graphite Showing Storage Capacity >330 mAh/g

**S**ample	BET surface area [m^2^/g]	Li+ discharge Capacity@0.1 C [mAh/g]	first Coulombic efficiency [%]	2 C charging vs 0.2 C charging [%]	3 C discharging vs 0.2 C discharging [%]
**Synthetic graphite**	1.4	338.8	94.5	19.7	96.3
**BDSG B**	7.6	331.0	88.3	25	59.7
**BDSG D**	9.2	335.7	87.2	26.5	68.6

The first cycle Li^+^ charge–discharge curves at a 0.1C rate are shown in Figure 14. From the charging curves, it is obvious that the biomass-derived graphite shows a lithium-ion insertion behavior very similar to that of the coke-based synthetic graphite. Specifically, the charging plateau below 0.2 V vs Li/Li^+^ indicates the Li^+^ insertion into the graphite interspace and the formation of the LiC_*x*_ substance. However, the biomass-derived graphite samples showed distinctly different behavior vs synthetic graphite from 0.4 to 0.6 V vs Li/Li^+^. At this voltage range, the reaction is mostly related to the decomposition of carbonate solvent on the surface of the carbon. In the future, more work could be done to reduce the electrolyte solvent decomposition. This will help to increase the reversibility of biomass-derived graphite BDSG as an anode active material. There are already industrial-scale options available to improve the surface compatibility, e.g., chemical vapor deposition of amorphous carbon.^24,59^

**Figure 14:**
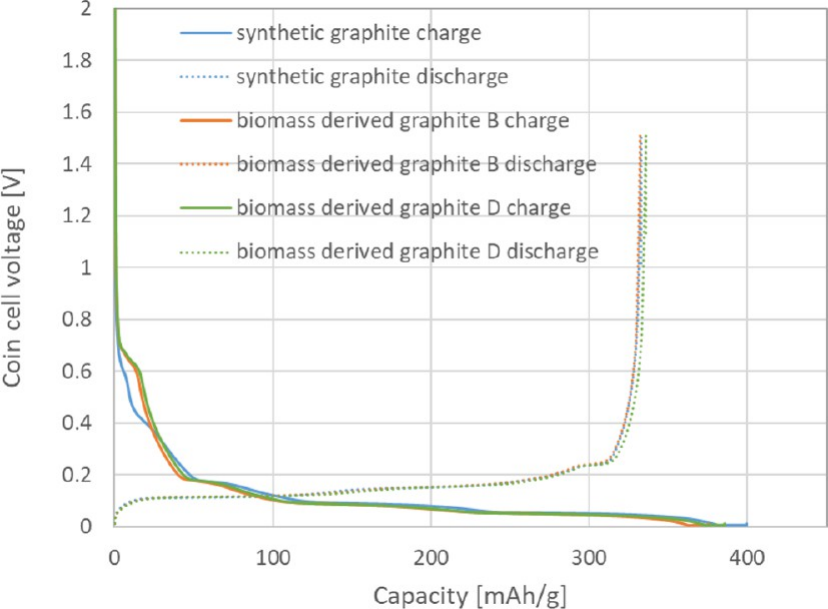
First cycle Li^+^ charge–discharge curves at a 0.1C rate of biomass-derived graphites BDSG B and D show similar Li^+^ ion insertion behavior as coke-based synthetic graphite.

The data in Table 5 are consistent with findings for other biobased graphites. Shi^19^ reports the electrochemical performance of catalytically prepared biobased anode material with a reversible capacity of 293 mAh/g at a current of 20 mA/g. Dey^56^ discloses a slightly better discharge capacity (350 mAh/g) and similar first Coulombic efficiency (90%), however, with significantly higher iron content (500 ppm) even after acid washing. Lower^22^ summarizes the electrochemical performance of catalytically graphitized biocarbons in a review with discharge capacities in the range of 204–340 mAh/g and reversible capacities between 27% and 84%. However, comparisons should be made cautiously due to variations in the preparation and measurement protocols.

## 5 Discussion

### 5.1 Sustainability Perspective

Biochar, a carbonaceous material derived from biomass pyrolysis, is attracting increasing attention for applications in carbon sequestration and environmental benefits. Life Cycle Assessment (LCA), standardized by ISO 14040 ^60^ and 14044,^61^ evaluates its environmental impact by inventorying inputs and releases, assessing impacts, and interpreting results. Climate Change, a key LCA indicator referring to the increase in average global temperatures as a result of Greenhouse gas (GHG) emissions, is constituted of three categories: Climate Change, fossil; Climate Change, biogenic; Climate Change, land use; and land use change.^62^ Modeling biogenic carbon is challenging due to the complexity and lack of harmonized methodologies in international guidelines and standards,^62,63^ which must be considered carefully when modeling them. Carbon, which exists in various forms, cycles between reservoirs into two primary time scales:

- Short-term cycles (less than a century) which include photosynthesis and soil respiration
- Long-term cycles (centuries) which include fossil fuel formation or peat decomposition.^64^

Biogenic carbon, derived from photosynthesis, belongs to the short carbon cycle, unlike fossil carbon.^65^ Two main approaches exist for accounting for biogenic carbon in LCA:^66^

- Carbon neutrality: This approach assumes equal CO_2_ inflow and outflow, resulting in a net-zero climate impact
- The −1/+1 approach: This method accounts for biogenic carbon similarly to fossil carbon by inventorying emissions. Besides, CO_2_ absorption from photosynthesis is credited. It is particularly suitable for biochar because it highlights the benefits of stabilizing carbon in a durable form.^64^

Biomass-derived graphite, produced through pyrolysis and graphitization, has effectively stabilized organic carbon for centuries. As an order of magnitude, a study by Fawzy et al.^67^ using LCA cradle-to-grave indicates that for every ton of biochar produced, 2.7 tCO_2_eq are permanently removed from the atmosphere. The −1/+1 accounting method allows for an accurate reflection of its environmental benefits. While LCA ISO standards mandate transparent methodological rules for biobased materials like biochar, methodological assumptions related to biogenic carbon modeling are rarely detailed. Key methodological variables requiring careful consideration include:^68,69^

- Biogenic carbon accountability: This requires clear modeling of fixed biogenic carbon and emissions to accurately reflect biochar’s carbon sequestration benefits
- Allocation methods: These are used to divide the environmental impact among the different outputs of the pyrolysis process, such as bio-oil, syngas, and biochar. Common approaches include system expansion or physical allocation based on the energy content of each product.
- Functional unit: The choice of the functional unit is vital for ensuring comparability across studies. It defines the basis for quantifying environmental impacts, such as per ton of pyrolyzed biomass or per unit of agricultural yield improvement
- System boundaries: Clearly defining system boundaries is crucial to specify what processes are included (e.g., feedstock cultivation, transportation, pyrolysis, and final application).

Beyond the modeling methodologies, several critical factors significantly affect the LCA outcomes of BDSG production:

- Feedstock selection: The type of biomass used as a feedstock (e.g., agricultural residues, forestry waste) directly impacts the biochar yield and its carbon sequestration potential, which is essential for determining an environmental performance^70^
- Pyrolysis process parameters: The pyrolysis process operates across a wide range of temperatures (300–700 °C) and time scales, which heavily influence the energy requirements and the properties of the resulting biochar. Higher temperatures often yield products with enhanced carbon stability but increase energy consumption^68,71^

- Graphitization process: The energy source and efficiency of converting biochar to graphite are crucial for the final carbon footprint as well as associated emissions.

Hence, methodological variables must be thoroughly considered to improve the reliability, transparency, and comparability of LCAs for biomass products. Biochar offers a promising alternative to fossil-based feedstocks in graphite production. BDSG, produced with comparable energy requirements, could significantly reduce the carbon footprint of applications such as batteries.

### 5.2 Formation Mechanism

As mentioned in the introduction section, there have been several successful studies in the past decade on the preparation of graphite from biomass or biocarbon via catalytic graphitization. Although the mechanism is still not completely understood, various authors propose possible mechanisms.^72,73,74^

However, catalytic mechanisms are not relevant to this study because, to our knowledge, graphite has been synthesized from biochar for the first time without the use of a catalyst. It is believed that as in the formation of natural or synthetic graphite, the role of volatiles is crucial in this case. However, it is not clear if the mechanism is the same as in the case of the formation of graphite from biological charcoal. This suggests that while volatiles contribute to the graphitization process of both soft carbons and hard carbons, the mechanism itself must be different. Mainly because of the absence of an important step: the formation of a carbonaceous mesophase in nongraphitizable carbons. Saavedra Rios et al.^75^ summarize in their review that the reason why hard carbon does not graphitize and does not have a long-range ordering along the *c*-axis is linked to its microstructure given by strongly cross-linked precursors. The cited models elaborate a theory based on the porosity and curvature of graphene/fullerene structures that prevent graphitization. Also Nair^15^ attributes the missing graphene stacking of biochar exposed to elevated temperature to the fact that the basic structural unit of the disordered carbon lies in a continuum between amorphous carbon and the defects containing graphite-like material. Prior to the graphitization of graphitizable carbon (e.g., coke), the carbonization process occurs where other important processes such as dehydrogenation, condensation, isomerization, and mesophase formation take place. The role of these processes and their difference are described in several handbooks.^6^ In comparison to cokes, chars are more branched, isotropic, and have a more turbostratic structure. This, together with their chemical composition, does not allow the same mechanism in the formation of graphite when exposed to high temperatures (e.g., no formation of mesophase, no dehydrogenation since it is already carbonized/pyrolyzed).

In the section below, we propose a mechanism for catalyst-free graphite formation from biomass-based feedstocks. It is suggested that volatiles, at least partially, take over the role of a catalyst (or radiation) and together with the internal stresses due to the high temperature contribute to the restructuring of carbonized char, followed by a precipitation of carbon and the formation of graphite. The importance of this step was also confirmed on fossil raw materials by González et al.^76^ by studying the ability of the anthracites to graphitize. It was found that the latter depends on the anisotropy of the texture of their carbonized form rather than on the anisotropy of raw anthracite. The role of volatiles and devolatilization in the changing of turbostratic structure and debranching is well known^77,78^ and also described for nongraphitizable carbon—coal by Manoj,^79^ where shortening of aliphatic chains is observed. Internal stress, together with volatiles, avoids the creation of curvature and porosity that is characteristic of hard carbons. The residues of this process are confirmed in this study by a rather low real (xylene) density compared to coke-based graphite, while having high crystallinity, as shown in XRD and Raman data. Of course, it should also be considered that biocarbon might be composed of fractions or domains of graphitizable (soft) carbons, where the mechanism would be similar to the graphitization of cokes. The mathematical model developed by Ouzilleau et al.^80^ suggests that all carbons, soft or hard, can be graphitized to some degree. On the other hand, the presence of these fractions cannot explain the results obtained in this study, showing a high graphitization degree reached without the use of a catalyst, which is contrary to other studies where only glassy carbon was obtained at the same temperature range.

To summarize, the proposed mechanism of biocarbon graphitization into graphitic forms is divided into these steps with increasing temperature:

- Temperatures above 1000 °C lead to biomass devolatilization and debranching of aromatics
- At the range of around 1700 °C–2000 °C creation of internal stress due to the sulfur, nitrogen, and volatiles release
- Instead of escaping, volatiles and aromatics anchor on the edge sites leading to crystal growth
- As a result, changing of the turbostratic structure into more ordered domains without curvature forming (cross-linking avoidance) occurs
- Stress release at 2500 °C or above results in graphene layer formation
- Restructuring graphene layer stacking into the graphite.

Above-mentioned mechanism is shown in Figure 15 for visualization purposes only.

**Figure 15:**
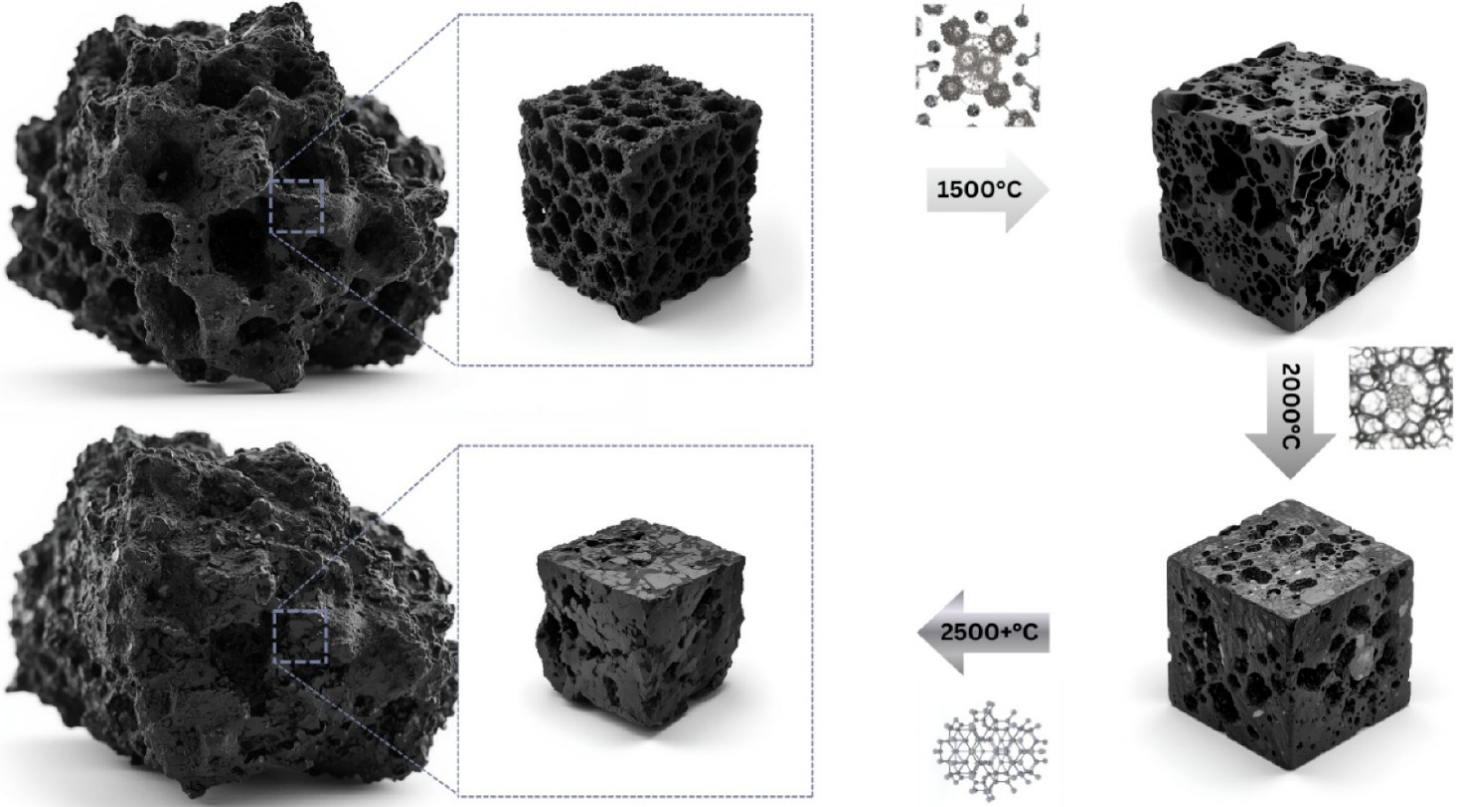
Schematic concept of the conversion of biomass-based material into synthetic graphite for visualization purposes (MG via Canva.com).

The authors of this study are aware that the term “volatiles” has a rather broad meaning. It will be the scope of follow-up research to understand in more depth the types of volatiles originating from coke vs biochar that are responsible for the above-mentioned mechanism.

## 6 Conclusion

Highly crystalline (*L_c_* > 100 nm) and high-purity (Fe < 50 ppm, Ni and Cr < 5 ppm, ash <0.1%) synthetic graphite with a graphitization degree of 91–95% was synthesized for the first time from renewable biomaterials without a catalyst, although generally considered as nongraphitizable under these conditions. Moreover, this was done at large, industrial scale using the Acheson process, a single-step synthesis process without the need for chemical purification. Further adjustment of the raw material and process parameters results in the same or better performance in applications such as polymer bipolar plates and carbon brushes. It is believed that additional coating with amorphous carbon, e.g., by chemical vapor deposition, will also lead to improved performance in lithium-ion batteries as an anode conductive active material.

This study also indicates directions on how to prepare CO_2_ neutral graphite or even carbon-negative graphite. From a sustainability perspective, stabilizing carbon from the short-term cycle into a durable form contributes to prolonged sequestration and reduced atmospheric carbon levels, while supporting defossilization goals.

## Supplementary Material



## Notes

Analytical methods description; Applicative test preparation and procedures (PDF)
