# Supplementary material for: High-Purity Biomass-Derived Synthetic Graphite: Catalyst-Free Industrial Synthesis and Applications
Source: ACS Omega. 2026 Jan 30;11(6):9435–50. doi: 10.1021/acsomega.5c09286 (PMC12917626; doi:10.1021/acsomega.5c09286)
Supplement: Supplementary file 1 [file ao5c09286_si_001.pdf]

SUPPORTING INFORMATION: High-Purity Biomass-Derived Synthetic Graphite: Catalyst-Free Industrial Synthesis and Applications

Michal Gulas[a,*], Flavie Delort[b], Raffaele Gilardi[a], Giovanni Juri[a], Luca Ostinelli[a], Frank Rauscher[a], Xu Wang[c], Simone Zürcher[a]

[a] - *IMERYS Graphite & Carbon, "Il Centro" Via Cantonale 65, 6804 Bironico, Switzerland*
[b] - *IMERYS, 43 Quai de Grenelle 75015 Paris , France*
[c] - *IMERYS Japan, E409 KSP 3-2-1 Sakado, Takatsu-ku, Kawasaki-city, Kanagawa 213-0012 Japan*
**- corresponding autho*r e-mail address: michal.gulas@imerys.com (Michal Gulas)

Analytical methods description; Applicative test preparation and procedures

Ash Content:
A low-walled ceramic crucible is heated to 810°C in a muffle furnace and cooled to room temperature in a desiccator. A sample of 10 g of dry powder (accuracy 0.1 mg) is weighed in the calcined crucible. The powder is combusted at a temperature of 810°C (at least 8 h) until a constant weight is reached. The residual corresponds to the ash content and is reported as a percentage of the initial weight of the sample.
Considering a typical moisture content of the sample ranging far below 0.5%, the values can be considered as measured on a dry sample. Regarding temperature, it is important to reach at least 750°C to allow for the ignition of the material to burn. ISO and ASTM normally have slightly different temperature ranges. It is important to ensure complete combustion while ensuring sufficient material remains for the ash content to be accurately weighed.
Normative reference:
DIN 51903 Testing of carbonaceous materials - Determination of ash value - Solid materials
Additional references:
ASTM C561 Standard Test Method for Ash in a Graphite Sample
ISO 1171 Solid Mineral Fuels – Determination of Ash ISO 6245 Petroleum Products – Determination of Ash
ISO 8005 Carbonaceous Materials Used in the Production of Aluminum – Green and Calcined Coke – Determination of Ash Content

Trace Elements – (SD Ar–OES):
Trace elements are assessed by Spark Discharge in Argon (SD Ar) and Optical Emission Spectrometry (OES). A graphite powder sample is ground to a maximum particle size of 75 µm by means of a vibrating mill and then pressed into a pill. The sample-pill is placed onto the excitation stand of the spectrometer. Under argon atmosphere spark discharges generate an emission of light, which is collected by the spectrometer. From the collected emission spectrum, the concentration of the different elements is calculated and then reported in mass-to-mass ratio expressed in ppm.
Reference:
K. Slickers, Automatic Emission Spectroscopy. Brühl Druck und Presshaus Giessen, 1992
Additional references:
ASTM E1999 Standard Test Method for Analysis of Cast Iron by Spark Atomic Emission Spectrometry
ASTM E1086 Standard Test Method for Analysis of Austenitic Stainless Steel by Spark Atomic Emission Spectrometry
ASTM E305 Standard Practice for Establishing and Controlling Spark Atomic Emission Spectrochemical Analytical Curves
DIN 51008 -1 Optical Emission Spectrometry (OES) – Part 1: Terms for Systems with Sparks and Low-Pressure Discharges

DIN 51009 Optical Atomic Spectral Analysis – Principles and Definitions

Interlayer Spacing c/2 and Crystallite Size Lc:

The crystalline structure is analyzed by means of an X-Ray Diffraction (XRD). A graphite powder sample is ground to a maximum particle size of 75 µm with a vibrating mill and is then mixed with a silicon standard. The mix is then placed in the sample holder in a thin layer of approximately 150 µm. The sample is irradiated using a Cu(K) X-ray beam at a different angle, the diffracted beam is collected by a detector. The angular position of the peak maximum of the (002) and (004) reflection profiles are determined and the interlayer spacing (c/2) is calculated by applying the Bragg equation. The crystallite size is determined by means of the Iwashita's algorithm, developed for carbon materials, and then by applying the Scherrer's equation. The calculated values are reported in nm. The results of c/2 and Lc are expressed in nm (nanometer, 1x10-9 m) or in Å (Ångström, 1x10-10 m) as they represent real dimensions of a crystal and not fractions. Therefore, neither of the crystallinity values are expressed as percentages (%) or similar expressions. The value of c/2 indicates the distance between the crystal plane.

Reference: N. Iwashita, C. Rae Park, H. Fujimoto, M. Shiraishi and M. Inagaki, Carbon, 42, 701-714 (2004)

Additional references:

ASTM D5187 Standard Test Method for Determination of Crystallite Size – Lc of Calcined Petroleum Coke by X-Ray Diffraction

ASTM D5380 Standard Test Method for Identification of Crystalline Pigments and Extenders in Paint by X-Ray Diffraction Analysis

ISO 20203 Carbonaceous Materials Used in the Production of Aluminium – Calcined Coke – Determination of Crystallite Size of Calcined Petroleum Coke by X-Ray Diffraction

Klug and Alexander, X-Ray Diffraction Procedures, John Wiley and Sons Inc., New York London (1967)

P. Scherrer, Göttinger-Nachrichten, 2, 98 (1918)

Xylene Density:

Xylene density is the short definition of the real density and erroneously called specific gravity, specific density or similar. The xylene density is obtained by xylene displacement. Approx. 2.5 g (accuracy 0.1 mg) of powder is weighed in a pycnometer. Xylene is added under a vacuum. After a few hours under normal pressure, the pycnometer is filled, conditioned and weighed. The density represents the ratio between the mass of graphite and the displaced volume of xylene. The values are reported in g/cm3.

Normative reference:

DIN 51901 Testing of Carbonaceous Materials – Determination of Density by the Xylene Method – Solid Materials

Additional references:

ASTM D5004 Standard Test Method for Real Density of Calcined Petroleum Coke by Xylene Displacement

ISO 8004 Carbonaceous Materials for the Production of Aluminium – Calcined Coke and Calcined Carbon Products – Determination of the Density in Xylene – Pyknometric Method

ISO 9088 Carbonaceous Materials Used for the Production of Aluminium – Cathode Blocks and Prebaked Anodes – Determination of the Density in Xylene – Pyknometric Method

Specific Surface Area by BET and Nitrogen Adsorption:

This measure is carried out by measuring the adsorption-desorption isotherm of nitrogen on the surface of the material by means of the Brunauer-Emmet-Teller (BET) algorithm. A definite amount (accuracy 0.01 mg) of powder is weighed in a sample tube. Then the sample undergoes a series of heating and cooling, from the evolution of the pressure in the sample tube the amount of absorbed nitrogen during the different steps is calculated; from this data the specific surface is calculated and reported in m2/g.

Normative reference: ISO 9277 Determination of the Specific Surface Area of Solids by Gas Adsorption – BET Method
Additional references:
ASTM B922 Standard Test Method for Metal Powder Specific Surface Area by Physical Adsorption
ASTM C1069 Standard Test Method for Specific Surface Area of Alumina or Quartz by Nitrogen Adsorption
ISO 15901-2 Pore Size Distribution and Porosity of Solid Materials by Mercury Porosimetry and Gas Adsorption – Part 2: Analysis of Mesopores and Macropores by Gas Adsorption
ISO 15901-3 Pore Size Distribution and Porosity of Solid Materials by Mercury Porosimetry and Gas Adsorption – Part 3: Analysis of Micropores by Gas Adsorption

<u>Spring-back:</u>
The spring-back is measured by compacting the graphite powders. Approximately 5 g (accuracy 0.1 mg) of graphite powder is poured into a die, then the punch is inserted and the air is evacuated. The sample undergoes a compression cycle up to a pressure of 0.47 ton/cm2 by which the height of the pill is collected under full pressure and after pressure released. The spring-back is the ratio between height difference and minimal height and is reported in %.
Additional references:
ASTM B331 Standard Test Method for Compressibility of Metal Powders in Uniaxial Compaction
ASTM B610 Standard Test Method for Measuring Dimensional Changes Associated with Processing Metal Powders
ISO 4492 Metallic Powders, Excluding Powders for Hard Metals – Determination of Dimensional Changes Associated with Compacting and Sintering
ISO 17172 Fine Ceramics (Advanced Ceramics, Advanced Technical Ceramics) – Determination of Compaction Properties of Ceramic Powders

<u>Apparent Density by Scott:</u>
The sample is free flowing through a Scott Volumeter and is collected in a one cubic inch vessel and weighed to 0.1 mg accuracy. The measure is performed three times and the average value is used. The ratio of average weight to volume (16.39 cm3) is the Scott density and is reported in g/cm3.
Normative reference:
ASTM B329 Standard Test Method for Apparent Density of Metal Powders and Compounds Using the Scott Volumeter
Additional references:
ISO 3923-2 Metallic Powders – Determination of Apparent Density – Part 2: Scott Volumeter Method

<u>Particle Size Distribution by Laser Diffraction:</u>
The Particle Size Distribution (PSD) for products ranging up to 150 µm is measured by means of the Laser Diffraction. A laser beam lights up the measuring chamber in which the graphite sample is blown by means of compressed air, the generated diffraction pattern is collected by means of a Fourier optic system and interpreted using standard models of the light scattering theory, such as that developed by Mie. The particle size distribution is calculated and reported in µm for the three (quantiles: 10% (d10), 50% (d50) and 90% (d90). For flaky graphite powders is necessary to consider that for such a material (irregular black grains and not perfect white spheres) the relative particle refractive index needs to be adapted to the applied light's wave numbers and instrument geometry. Experience suggests a range around 1.15 for the HeNe's red (633 nm).
Normative reference:
ISO 13320 Particle Size Analysis Laser Diffraction Methods
Additional references:
ASTM B822 Standard Test Method for Particle Size Distribution of Metal Powders and Related Compounds by Light Scattering

ASTM D4464 Standard Test Method for Particle Size Distribution of Catalytic Material by Laser Light Scattering
ASTM E1458 Standard Test Method for Calibration Verification of Laser Diffraction Particle Sizing Instruments using Photomask Reticles
ASTM E2651 Standard Guide for Powder Particle Size Analysis
ISO 8130 -13: Coating Powders – Part 13: Particle Size Analysis by Laser Diffraction
ISO 14887 Sample Preparation – Dispersing Procedures for Powders in Liquids

<u>Particle Size Distribution by Vibrating Sieving:</u>
The measure is performed using a pile of sieves with descending mesh size on a sieving machine. 100 g (accuracy 0.01 g) of graphite sample is poured onto the top screen, then a vibration cycle is started. The residue on the screens and in the bottom pan is weighed and the particle size distribution (PSD) is calculated and reported in % as cumulative starting from larger particles.
Normative references:
DIN 66165-1 Particle size analysis - Sieving analysis - Part 1: Fundamentals
DIN 66165-2 Particle size analysis - Sieving analysis - Part 2: Procedure
Additional references:
ASTM D4749 Standard Test Method for Performing the Sieve Analysis of Coal and Designating Coal Size
ASTM E2651 Standard Guide for Powder Particle Size Analysis
ASTM MNL32 Manual on Test Sieving Methods ASTM STP447A Manual on Test Sieving Methods
DIN 51938 Testing of carbonaceous materials - Determination of particle size distribution by sieving - Solid matters
ISO 1953 Hard Coal – Size Analysis by Sieving ISO 4497 Metallic Powders – Determination of Particle Size by Dry Sieving

<u>Volatile Matter:</u>
A suitable amount of pre-dried (125°C) sample is weighed in a proper vessel and placed inside a nitrogen flushed tubular furnace. The furnace is heated to 950°C for 30 minutes. After cooling in a nitrogen atmosphere, the loss of weight of the sample is determined and expressed as a percentage of the initial weight of the sample.

<u>Carbon Brush testing:</u>
Sample preparation: dry mix of 80wt% graphite powder with 20wt% of phenolic resin powder (Supraplast 101/3040 by Süd-West Chemie GmbH)) have been pressed at 2 t/cm2 and 4 t/cm2 and heat-treated.
The respective graphitic materials (80 wt.%) were mixed with a phenolic resin at about 20°C by a Stuart roller mixer using steel balls (model number SRT9D) for 30 min at 60 rpm to form polymer/graphite mixtures. For each of the polymer/graphite mixtures, a 5 gram quantity of the prepared polymer/graphite mixture was pressed at room temperature with two different pressures (2 t/cm2 and 4 t/cm2, respectively) in a press, model number PW 40 EH-PRESSYS by Paul-Otto Weber GmbH, having a die of 50x12 mm2 to form green plates with thickness of about 5 mm. The green plates were further reticulated by heating for the following ramped times and temperatures to obtain the final phenolic resin composites:
ramped from a temperature of 25°C to 80°C over a period of 120 minutes; then
ramped from a temperature of 80°C to 135°C over a period of 660 minutes; then
ramped from a temperature of 135°C to 180°C over a period of 270 minutes; then
kept at 180°C for 120 minutes; then followed by cooling to room temperature over a period of 120 minutes.
Electrical resistivity has been measured using Schuetz MR1012S by four-point method in the "in-plane" and "through-plane" directions and results are plotted as a function of the density of the carbon brush.

The configuration used for this measurement is a planar stack composed of 2 metal plates as current electrodes, 2 sheets of graphite (intermediate layer of contact), the sample sandwiched between the graphite sheets, and 2 thin gold wires (diameter = 0.125 mm) as measuring electrodes positioned between the sample and the graphite foils. A pressure of 3 N/mm2 is applied to the stack to reduce contact resistances through a press.
Electrical resistance is measured using Schuetz MR1012S and electrical resistivity is calculated according to the formula
$\rho = R*(L*W)/t$
*where ρ is the resistivity in Ω*cm, R is the resistance in Ohms, t is the thickness of the sample, L is the length of the specimen and W is the width of the specimen.*

<u>Bipolar plates</u>
Sample preparation: polymer compounds with 80wt% graphite and 20wt% polypropylene homopolymer (Moplen HP501L by LyondellBasell) were prepared in the internal mixer (HAAKE Rheomix 600 OS). Graphite was mixed with polypropylene for 5 min at 220 °C and 100 rpm.
60x60x2 mm3 plates were prepared by compression molding using a LabTech Scientific LP-S-20 and samples (50x12x2 mm3) were cut for resistivity measurements (done with same setup as for carbon brush).

<u>Anode active material for LiB</u>
Preparation of the negative electrode slurries
Negative electrodes for use were prepared from reference anode active material (graphite), biomass derived graphite & carbon black (Imerys Graphite & Carbon C65T), carboxymethyl cellulose (Nippon Seishi MAC500LC), an aqueous Styrene-Butadiene Rubber (SBR, 40 wt. %, Zeon), using a Primix 2P-03 type mixer. The final slurry composition for the negative electrode was graphite or biomass derived graphite: carbon black: CMC: SBR in a 96.5:1:1.5:1 ratio respectively, with a solid content of 50 wt.% based on the total weight of the electrode slurry. The solvent used in negative electrode preparation was DI-water. The slurry was coated onto a 18 um Cu foil with a bar coater. The slurry was dried in an oven preset at 80 degrees. The average loading mass of graphite or biomass-based graphite per electrode was 9 mg/cm2. The electrodes were pressed by a calendaring machine to a density of 1.5 to 1.55 g/cm3 for the electrochemical evaluation.

Coin Cell Assembly
CR2032 type coin cells were prepared to check the electrochemical performance of different samples. A piece of Li metal was used as both the counter and reference electrode. The electrolyte was 200µl 1M LiPF6 EC/ECM/DMC (3/5/2 in weight). The separator used was a piece of Celgard ® 2500 monolayer microporous membrane (obtainable from Celgard LLC).

Coin cell test protocol
For the Li^+ discharge rate test of coin cells, coin cells were first discharged at a constant current of 0.2C (1C=372 mAh/g) to 0.005V, then the voltage was maintained at 0.005V until the current dropped to 0.01C. The cell was then charged at a constant current of 3C. The Li^+ discharge capacity retention percentage at 3C is the ratio between constant current charged capacities at 3C versus 0.2C. For the Li^+ charge rate test of coin cells, coin cells were discharged at 2.0C to 0.005V and at a constant current, then the cell voltage was maintained at 0.005V until the current dropped to 0.01C. The cell was then charged at 0.2C. The Li^+ charge rate performance percentage at 2C rate is the ratio between constant current charged capacities at 2C versus 0.2C. The coin cell testing is performed using a charge-discharge cycler, model number TOSCAT-3100, obtained from Toyo system Co. Ltd.
